# Drought-Responsive Mechanisms in Plant Leaves Revealed by Proteomics

**DOI:** 10.3390/ijms17101706

**Published:** 2016-10-18

**Authors:** Xiaoli Wang, Xiaofeng Cai, Chenxi Xu, Quanhua Wang, Shaojun Dai

**Affiliations:** Development Centre of Plant Germplasm Resources, College of Life and Environmental Sciences, Shanghai Normal University, Shanghai 200234, China; wangxl@shnu.edu.cn (X.W.); xfcai@shnu.edu.cn (X.C.); chenxixu@shnu.edu.cn (C.X.); wangquanhuan@shnu.edu.cn (Q.W.)

**Keywords:** proteomics, leaves, drought stress, molecular mechanism

## Abstract

Plant drought tolerance is a complex trait that requires a global view to understand its underlying mechanism. The proteomic aspects of plant drought response have been extensively investigated in model plants, crops and wood plants. In this review, we summarize recent proteomic studies on drought response in leaves to reveal the common and specialized drought-responsive mechanisms in different plants. Although drought-responsive proteins exhibit various patterns depending on plant species, genotypes and stress intensity, proteomic analyses show that dominant changes occurred in sensing and signal transduction, reactive oxygen species scavenging, osmotic regulation, gene expression, protein synthesis/turnover, cell structure modulation, as well as carbohydrate and energy metabolism. In combination with physiological and molecular results, proteomic studies in leaves have helped to discover some potential proteins and/or metabolic pathways for drought tolerance. These findings provide new clues for understanding the molecular basis of plant drought tolerance.

## 1. Introduction

Drought is an inevitable and recurring feature of world climate. Despite our efforts to forecast its onset and modify its impact, drought remains the single most important factor affecting worldwide crop growth and productivity [1]. More importantly, although plant drought response has been extensively studied [2,3,4], there are still no economically practical technological means to facilitate crop production under drought [5]. Therefore, there is an urgent need to further understand and enhance crop tolerance to drought stress.

Drought stress induces a number of changes at the morphological, physiological and biochemical level in all plant organs [6]. Plants have evolved several strategies to cope with drought stress, including drought escape via a short life cycle or developmental plasticity, drought avoidance via enhanced water uptake and reduced water loss, as well as drought tolerance via osmotic adjustment, antioxidant capacity, and desiccation tolerance [7]. With the development of high-throughput sequencing and various omic technologies, large amounts of sequence data sets and global changes in gene expressions have been reported. For example, using genomics and quantitative trait loci (QTL) mapping approaches, a number of major genes or QTL for drought stress resistance were identified in *Arabidopsis* [8], pearl millet (*Pennisetum glaucum*) [9], *Festuca pratensis* (Huds.) [10], wheat (*Triticum aestivum*) [11], maize (*Zea mays*) [12], and common bean (*Phaseolus vulgaris*) [13]. Besides, microarray analysis of drought response in shoots of two *T. aestivum* cultivars (i.e., *TAM 111* and *TAM 112*) have revealed that 1657 transcripts were commonly altered in both cultivars, but 474 and 1540 transcripts were unique to each cultivar, respectively [14]. In addition, genome-wide transcriptional analysis of *Populus euphratica* under four different drought treatments (watering with 70%, 50%, 20%, and 5% of daily evaporation for seven weeks) have identified 952, 1354, 2138 and 2360 transcripts, respectively. Among them, some candidate drought-responsive genes have a high potential to be used for crop breeding with drought tolerance. Those genes are involved in the biosynthesis of plant hormones, various signaling pathways (e.g., *ZEP*, *PYL*, *PP2C*, *SnRK2*, *ACO*, *ACS*, *ETR1*, and *ETO1*), osmoprotective pathways (e.g., *BADH*, *P5CS*, *PDH1*, *TPS1*, and *LEA*), as well as the detoxification of reactive oxygen species (e.g., *APX*, *SOD*, *GR*, and *ALDH7*) [15].

**Figure 1:**
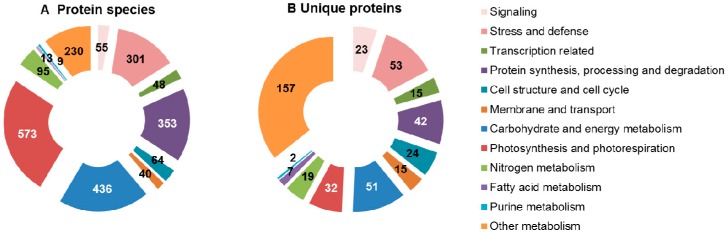
Functional categories of drought-responsive protein species and unique proteins in leaves revealed by proteomic studies. (**A**) Protein species include all the protein isoforms generated from gene variable splicing and post-translational modifications in the original publications; (**B**) Unique proteins indicate the protein family whose members have similar protein sequence homology and functional domains.

However, the mRNA levels usually do not correlate well with the protein abundances and functions, due to various post-translational modifications. High-throughput proteomics has proved to be a powerful tool for the comprehensive identification of drought-responsive proteins in plants [16,17]. In previous investigations, more than 2200 drought-responsive protein species have been identified in leaves from 25 plant species, including 18 herbs, 3 shrubs and 4 trees (Table 1, Supplementary Table S1). In the present review, these drought-responsive protein species are defined as 440 unique proteins on the basis of their protein sequence homology and functional domain similarity. These proteins are mainly involved in signaling, transcription, stress and defense, protein synthesis, folding and degradation, photosynthesis and photorespiration, carbohydrate and energy metabolism, membrane and transport, cell structure and cell cycle, nitrogen assimilation and amino acid metabolism, as well as fatty acid metabolism (Figure 1). In addition, we also found some drought-responsive phosphoproteins in four phosphoproteomics studies (Table 1, Supplementary Table S2). Phosphorylation is one of the most important post-translational modifications (PTMs) that modulates protein activity, protein–protein interaction and cellular localization. These drought-responsive phosphoproteins were mainly involved in signaling, transcription, photosynthesis and carbon metabolism, as well as in protein synthesis and turnover. The integrative analysis of physiological, molecular, and proteomic characteristics provides new clues for further understanding plant drought tolerance.

## 2. Drought Sensing and Signaling

### 2.1. Possible Drought Receptor

**Figure 2:**
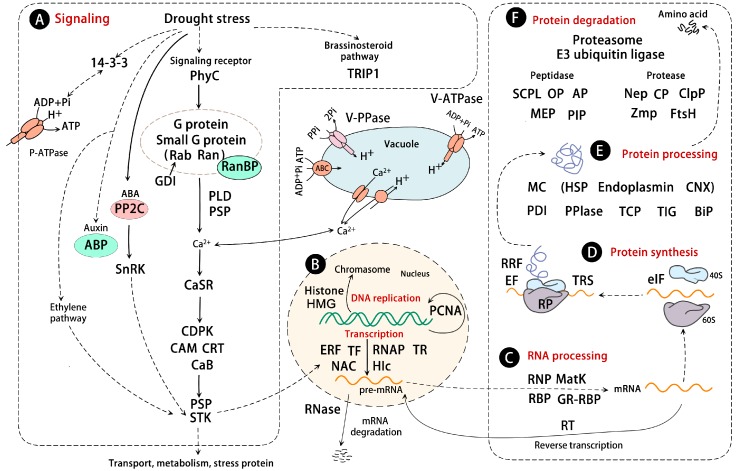
Schematic representation of drought responsive proteins involved in signaling, gene expression regulation, protein synthesis and degradation. The solid line indicates single-step reactions, and the dashed line indicates multi-step reactions. (**A**) Signaling; (**B**) DNA replication and transcription; (**C**) RNA processing; (**D**) Protein synthesis; (**E**) Protein processing; (**F**) Protein degradation. 14-3-3: 14-3-3 protein; Nep: aspartic proteinase; ClpP: ATP-dependent Clp protease; ABP: auxin-binding protein; BiP: endoplasmic reticulum-luminal binding protein; CaB: calcium ion binding protein; CaSR: calcium sensing receptor; CDPK: calcium-dependent protein kinase; CAM: calmodulin; CNX: calnexin; CRT: calreticulin; CP: cysteine proteinase; EF: elongation factor; ERF: ethylene-responsive transcription factor; GDI: GDP dissociation inhibitor; GR-RBP: glycine-rich RNA binding protein; HSP: heat shock protein; HMG: high mobility group protein; HOP: Hsp70-Hsp90 organizing protein; AP: leucine aminopeptidase; MatK: maturase K; MEP: metalloendopeptidase; MC: molecular chaperone; OP: oligopeptidase A-like; PPIase: peptidyl-prolyl *cis*-*trans* isomerase; PLD: phospholipase D; PhyC: phytochrome C; PCNA: proliferating cell nuclear antigen; PIP: proline iminopeptidase, putative; PDI: protein disulphide isomerase; PP2C: protein phosphatase 2C; RanBP: Ran-binding protein; RT: retrotransposon protein; RNase: ribonuclease; RNP: ribonucleoprotein; RP: ribosomal protein; RBP: RNA binding protein; Hlc: RNA helicase; RNAP: RNA polymerase; SCPL: serine carboxypeptidase-like protein; STK: serine/threonine kinase; PSP: serine/threonine-protein phosphatase; SnRK: sucrose non-fermenting 1-related protein kinase; TCP: T-complex protein; TRIP1: TGF-β receptor-interacting protein 1; TF: transcription factor; TR: transcription regulator; TIG: trigger factor-like protein; V-PPase: vacuolar H^+^-pyrophosphatase; V-ATPase: vacuolar H^+^-ATPase; Zmp: zinc metalloprotease.

Although no specific receptors for drought sensing have been found in plants, a drought-responsive photoreceptor, phytochrome C1, was identified in *Z. mays* [41] (Figure 2A). Phytochrome is widely believed to regulate the transcription of light-responsive genes by modulating the activity of several transcription factors under both biotic and abiotic stresses [65]. In *Arabidopsis*, three phytochrome genes (i.e., *PHYA*, *PHYB*, and *PHYE*) are involved in suppressing drought tolerance [66]. These results imply a possible function of phytochrome C in mediating osmotic stress.

### 2.2. G Proteins

G proteins constitute one of the most important cell signaling cascades. Proteomic studies revealed that two G protein subunits (alpha subunit and beta subunit), several small G proteins (e.g., Ras-related protein Rab7 and Ras-related nuclear protein Ran), and a Ran-binding protein 1 were increased in drought-treated leaves (Supplementary Table S1, Figure 2A). Their functions in drought tolerance have been previously reported. The G protein alpha subunit may play a positive role in regulation of drought stress [67,68], whereas the G protein beta subunit in *Arabidopsis* may negatively regulate drought tolerance [69,70]. Besides, Rab proteins are the largest branch of the small G protein superfamily, which are involved in vesicle trafficking, intracellular signaling events and many important physiological processes, such as polar growth, plant hormone signal cross-talk, and stress response [71]. One of the Rab proteins, Rab 7, has been suggested to be involved in drought stress tolerance. The *PgRab7* gene was upregulated by dehydration in *P. glaucum* [72], while overexpression of the peanut *AhRabG3f* exhibited an enhanced tolerance to drought stress in transgenic peanut (*Arachis hypogaea* L.) [73]. Additionally, two isoforms of GDP dissociation inhibitor (GDI), involved in the regulation of Rab family activity, were increased in drought-stressed leaves of *Oryza* *sativa* [29]. The expression of the *MiRab-GDI* gene was induced in *Mangifera indica* under drought stress conditions [74], but the exact function of GDI in response to drought stress remains to be elucidated. In addition, Ran is another member of the small G protein group, which is involved in nucleo-cytoplasmic transportation of proteins and RNA, the formation of spindle asters, and the reassembly of the nuclear envelope in mitotic cells [71]. Ran-binding protein 1 is the major effector of Ran. The significant increase of Ran in *Poa pratensis* [23] and Ran-binding protein 1 in *Z. mays* under drought stress conditions [41] indicated the important role of cell cycle and DNA synthesis in drought tolerance. However, the drought-decreased Ran/TC4 in *Cistus albidus* under no irrigation for 107 days [53] implies their function is dependent on the drought intensity.

### 2.3. Ca^*2+*^ Signaling and Protein Kinase

Under drought stress, calcium acts as a second messenger, which is employed to regulate specific protein kinase activity and downstream gene expression [75]. In proteomic studies, the abundances of several calcium binding proteins (CaBs), such as calmodulin (CaM), calcium sensing receptor (CaSR), calreticulin (CRT), and calcium-dependent protein kinase (CDPK), were changed in response to drought (Figure 2A). Among them, CRT is a unique endoplasmic reticulum (ER) luminal Ca^2+^ binding chaperone, which plays a role in many cellular processes [76]. The increase of CRT abundance enhanced the survival of *T. aestivum* plants under drought condition [77], and the *TaCRT*-overexpressing tobacco (*Nicotiana benthamiana*) plants exhibited enhanced drought resistance [78]. Proteomic studies revealed a drought-induced CRT1 in *Glycine* *max* [17], but a drought-reduced CRT1 in *Quercus* *robur* under prolonged (65 days) drought stress [61]. These results indicate that CRT functions in drought tolerance in a stress intensity-dependent manner. Besides, CDPKs regulate the downstream components in calcium-mediated signal transduction. In proteomic studies, the protein abundance and phosphorylation level of CDPKs were generally decreased in plants under drought. However, the abundance of CDPKs increased in the drought-tolerant genotype of *Z. mays* [41]. It has been reported that overexpression of the *CDPK* gene can enhance drought tolerance in transgenic *Arabidopsis* [79,80] and *O. sativa* [81]. These reports imply the important role of CDPKs in plant stress tolerance. The phosphorylation states of several protein kinases (e.g., serine/threonine-protein kinase, germinal center kinase (GCK)-like kinase MIK, receptor-like protein kinase HERK 1-like, phototropin family protein kinase, and salt-inducible protein kinase) were also changed in response to drought stress, implying their regulation in the drought response signaling pathway. In addition, the dephosphorylation mediated by protein phosphatases is an important event in the signal transduction process that regulates various cellular activities [82]. In proteomic studies, the phosphorylation level of protein phosphatase 2C (PP2C) and phospholipase D (PLD) in *Z. mays* [41] were drought-increased (Figure 2A). PP2C is known to be a negative regulator for plant drought tolerance in the abscisic acid (ABA) signaling pathway, which can inhibit the activity of SnRK, leading to a decrease of the phosphorylation of its substrates in the signaling cascade [83,84,85]. Both phosphorylation level [40,44] and abundance of PP2C [41,86] were obviously affected by drought stress. These data suggest that the PP2C-involved ABA signaling pathway is crucial for drought response. Additionally, a dual function of PLD in plant drought response was reported, for example, in drought-sensitive cultivars, the activity and expression of PLD increased more obviously than in drought-tolerant ones [87,88], whereas overexpression of the *PLDα* gene can significantly enhance drought tolerance in transgenic *Arabidopsis* [89] and *Populus tomentosa* [90]. In addition, a high level of PLD promotes stomatal closure at earlier stages, but disrupts membranes in prolonged drought stress [91]. These data imply that, in drought, PLD functions in a condition-dependent manner.

### 2.4. 14-3-3 Proteins

In proteomic studies, some members of the 14-3-3 protein family and 14-3-3-like proteins were increased in drought-treated leaves from many plant species (Figure 2A, Supplementary Table S1). The 14-3-3 proteins are intracellular dimeric phosphoserine/threonine binding molecules that participate in a wide range of vital regulatory processes, including signaling, transmembrane receptors, and transcription activation [92]. 14-3-3 proteins are also known as positive regulators of H^+^-ATPase activity to control the electrochemical gradient across the plasma membrane, which contributes to the initiation of stress responses and other signal transduction pathways [93]. It has been reported that drought stress can directly alter the abundance of 14-3-3 proteins [94,95,96]. In addition, overexpression or silencing of the 14-3-3 protein genes can modulate drought tolerance of transgenic plants (e.g., *Gossypium hirsutum* and *Arabidopsis*) [97,98]. These results imply that 14-3-3 proteins may have diverse regulatory roles in leaves under drought stress, and the involvement of each 14-3-3 protein in drought-response needs to be further elucidated.

### 2.5. Ethylene and Auxin Signaling Pathways

It is well known that ABA is a key phytohormone that mediates the adaptive response to drought stress. Besides, other hormonal signals, such as ethylene, gibberellic acid, and jasmonic acid are also important cellular regulators in signal transduction pathway under drought conditions [99,100,101]. Proteomic studies revealed a drought-increased ethylene-responsive transcription factor (ERF) in *Gossypium* *herbaceum* [51] and some members of drought-responsive auxin-binding protein (ABP) family in *Q. robur* [61], *Z. mays* [41], and polar clones [59]. The *ERF* gene was found to be induced in *G. herbaceum* under drought stress [102,103] (Figure 2A). Overexpression of *ERFs* in various plants, such as sugarcane *SodERF3* overexpression in tobacco, tomato *TERF1* in rice, and *Brassica rapa BrERF4* in *Arabidopsis*, can improve plant drought tolerance. However, careful attention should be taken in the definition of ERF roles in plant drought tolerance. Different ERF members may play different roles in plants in response to drought stress. For example, *BpERF11* was found to negatively regulate osmotic tolerance in *Betula platyphylla* [104]. In addition, ABP functions as an auxin receptor, being involved in many development processes and drought response. However, as far as we know, little information is available for ABP members (i.e., ABP2, ABP20, and ABP19a) in response to drought stress. Additionally, a drought-increased TGF-β-receptor interacting protein 1 (TRIP1) was found in *Sporobolus* *stapfianus* [24]. TRIP-1 was phosphorylated by the brassinosteroid (BR)-insensitive I (BRI-1) protein, which is a serine/threonine kinase receptor essential for BR perception and signal transduction. The increase of TRIP-1 in *S. stapfianus* suggests that the BR signaling pathway would be triggered in response to water deficit.

## 3. Drought-Responsive Gene Expression Regulation

Drought-responsive gene expression is crucial in the transcriptional regulatory network [105]. Proteomic studies have found some gene expression-related proteins in the complex regulatory networks in leaves (Figure 2B). Chromatin structure modification is a prerequisite to sustain the transcriptional regulation which is involved in cell cycle progression. Significant abundance changes were observed in two chromatin structure modification-related proteins, which were histones and high mobility group proteins (HMG). Histones are the major proteins of chromatin, and the dynamic association of histones and their variants can regulate gene expression [106]. In proteomic studies, several histones (e.g., H1 and H2B) appeared to cause diverse abundance changes in different plant species in response to drought stress. For example, H2Bs were decreased in *C. albidus* [53] and *Brassica* *napus* [47], while histone H1 was decreased in a drought-sensitive *Z. mays* cultivar, but increased in a drought-tolerant one [41]. Similarly, the transcript and protein of histone H1 variant were all induced specifically in the tolerant genotype of *G. herbaceum* [107]. Besides, HMG is a highly conserved nuclear DNA-binding protein, which functions in DNA repair and chromatin modification after DNA damage [108]. A proteomic study revealed that two isoforms of HMG were drought-increased in drought-tolerant maize cultivar, but decreased in a sensitive cultivar [41]. Interestingly, the phosphorylation level of HMG was significantly decreased in a drought-tolerant wheat cultivar, but increased in a drought-sensitive one [40]. Previous studies have reported that phosphorylation of HMG reduced its binding to DNA, inhibiting replication and transcription [109]. These data imply that PTM (e.g., phosphorylation) of HMG is crucial for its function in plant drought tolerance.

In addition, RNA processing was also critical for plants to cope with drought stress. The abundances of several RNA processing-related proteins were changed in plants under drought stress (Figure 2C). Among them, five glycine-rich RNA binding proteins (GR-RBPs) were drought-increased, while three GR-RBPs were drought-decreased. RNA binding proteins (RBPs) can bind to RNA molecules, which are involved in almost all aspects of post-transcriptional gene regulation. The *GR-RBP* genes have been noted to be upregulated in response to water stress [110], which was suspected to function in the regulation of specific gene expression in response to stress. For example, the expression of *GR-RBP* gene in transgenic rice has been shown to have much higher recovery rates and grain yields when compared with that in wild-type plants under drought conditions [111]. However, the transgenic *Arabidopsis* and camellia plants that had an overexpressed *GR-RBPa* gene from *Camelina sativa* appeared to have a reduced drought tolerance [112]. Besides, proteomic studies revealed that the abundance and phosphorylation level of S-like ribonucleases (RNases) were significantly increased in rice under drought stress [30,34]. S-like RNases have acquired specialized functions such as stress regulation, defense against microorganisms, phosphate scavenging, and even nitrogen storage [113]. It has been reported that S-like RNases participated in the responses to salt, polyethylene glycol (PEG), and ABA [113]. All these have attracted great attention as researchers have attempted to further confirm their post-transcriptional regulation in response to drought stress. Additionally, an intron splicing related protein, and maturase K (MatK) appeared to show a drought decrease in poplar [60], and multiple organelle RNA editing factor 9, involved in RNA editing in mitochondria and plastids, was increased firstly, and then decreased in *B. napus* with the extension of drought stress [47]. Both of them are RNA processing-related proteins. Their changes indicated that the transcription regulation is diverse and complicates the study of plants’ ability to cope with drought stress.

## 4. Drought-Responsive Protein Synthesis and Turnover

Protein synthesis and turnover is one of the fundamental metabolic processes for plants to cope with drought stress. Proteomic investigations revealed that 16% of the drought-responsive proteins in leaves are attributed to protein synthesis and turnover functions (Figure 1). Several proteins are involved in protein biosynthesis, such as ribosomal protein (RP), elongation factor (EF), translation initiation factor (TIF), tRNA synthase (TRS), and ribosome recycling factor (RRF) (Figure 2D). Most of them exhibited an increase under drought stress, which would be beneficial for protein synthesis in response to specific drought conditions. Besides, the proteins functioning in protein folding and processing showed diverse changes among different plant species and cultivars (Figure 2E). For example, peptidyl-prolyl *cis*-*trans* isomerases (PPIases) were significantly increased in *G. max* [17], *T. aestivum* [35], *O. sativa* [29], and *Q. robur* [61], but decreased in a drought-sensitive cultivar of *Phaseolus* *vulgaris* [49]. Protein disulfide isomerases (PDIs) were increased in barley and *B. napus*, but decreased in *Agrostis* *stolonifera*, *Q. robur*, and poplar. Additionally, ER-luminal binding protein (BiP), trigger factor-like protein (TIG), most heat shock proteins (HSPs), and other molecular chaperones (i.e., calnexin, endoplasmin) were increased, but T-complex protein and HSP70-HSP90 organizing protein were decreased in drought-treated leaves (Supplementary Table S1). These proteins function to maintain normal protein folding, repairing, and renaturation of the stress-damaged proteins. Among them, HSPs function in protein folding for drought tolerance, which has been widely discussed [114,115,116]. *HSP* genes have been transferred into *Arabidopsis* and yeast to improve their drought tolerance [117,118]. These data suggest that maintaining correct protein folding is important for leaves to cope with drought stress [35].

Besides, protein degradation is important to remove abnormal or damaged proteins and to control the levels of certain regulatory proteins during drought stress. Proteomic studies also revealed that some protein degradation-related proteins increased in response to drought stress, including proteasomes, proteases, and peptidases (Figure 2F). Previous studies have reported that some components in the protein degradation pathway, such as ubiquitin/26S proteasomes, small ubiquitin-like modifier (E3 SUMO) ligase, and proteases/peptidases were involved in plant drought tolerance [119,120,121,122,123]. For the ubiquitin/26S proteasome system, 7 out of 11 20S proteasomes (the core regulatory particle of 26S proteasome) were increased in leaves of *P. vulgaris* [49], *Hordeum* *vulgare* [26], *B. napus* [47], and *Medicago* *sativa* [50], respectively. Importantly, the phosphorylation level of E3 ubiquitin ligase, which is one of the key enzymes involved in ubiquitination, exhibited significantly increased values in leaves under drought stress [40,44]. Many studies have shown that E3 ubiquitin ligases were positively related to plant drought tolerance [124,125,126]. These findings indicate that the enhancement of the ubiquitin/26S proteasome system is important for plants to cope with drought. In addition, most proteases were drought-increased in plants, such as ATP-dependent Clp protease in *H. vulgare*, cysteine proteinase in *P. vulgaris*, zinc metalloprotease in *B. napus*, and aspartic proteinase in *Z. mays*. Consistently, some peptidases, such as serine carboxypeptidase in *G. herbaceum* and oligopeptidase in *O. sativa*, were increased in drought-stressed leaves. Among them, aminopeptidases (APs), catalyzing the hydrolysis of amino acids from the N-terminus of proteins, were generally increased in drought-tolerant plant species, and decreased in drought-sensitive plant species or cultivars. The essential role of APs in plant drought tolerance has been well addressed previously [123].

## 5. Reactive Oxygen Species (ROS) Scavenging Pathways

### 5.1. Superoxide Dismutase (SOD), Catalase (CAT), and Peroxidase (POD) Pathway

**Figure 3:**
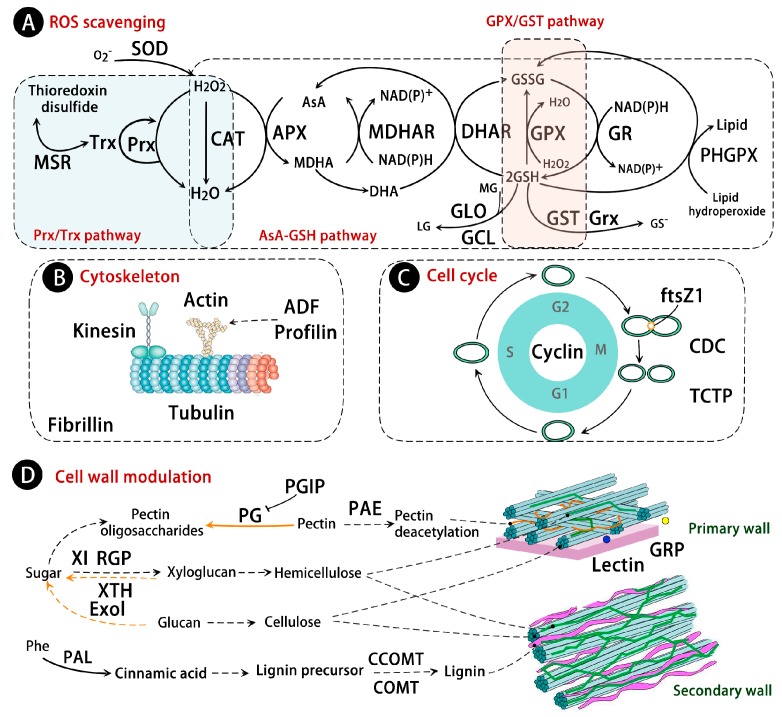
Schematic representation of drought responsive proteins involved in reactive oxygen species (ROS) scavenging, cell structure and cycle. The solid line indicates single-step reactions, and the dashed line indicates multi-step reactions. (**A**) ROS scavenging; (**B**) Cytoskeleton; (**C**) Cell cycle; (**D**) Cell wall modulation. ADF: actin depolymerizing factor; ALDH: aldehyde dehydrogenase; AsA: ascorbate; APX: ascorbate peroxidase; Exol: glucan exohydrolase; COMT: caffeic acid 3-*O*-methyltransferase; CCOMT: caffeoyl-CoA *O*-methyl-transferase; CAT: catalase; CDC: cell division cycle protein; DHAR: dehydroascorbate reductase; DHA: dehydroascorbate; GME: GDP-d-mannose-3′,5′-epimerase; GCL: glutamate-cysteine ligase; Grx: glutaredoxin; GPX: glutathione peroxidase; GR: glutathione reductase; GST: glutathione *S*-transferase; GSSG: glutathione disulfide; GSH: glutathione; GRP: glycine-rich protein; GLO: glyoxalase; LG: (*R*)-*S*-lactoylglutathione; MSR: methionine sulfoxide reductase; MG: methylglyoxal; MDHAR: monodehydroascorbate reductase; MDHA: monodehydroascorbate; ftsZ1: plastid division protein ftsZ1 precursor; PAE: pectin acetylesterase; PAL: phenylalanine ammonia-lyase; PG: pectin depolymerase; PGIP: polygalacturonase inhibitor; PHGPX: phospholipid hydroperoxide glutathione peroxidase; POD: peroxidase; Prx: peroxiredoxin; RGP: reversibly glycosylated polypeptide; SOD: superoxide dismutase; Trx: thioredoxin; TCTP: translationally-controlled tumor protein homolog; XI: xylanase inhibitor; XTH: xyloglucan endotransglycosylase.

Water deficit interrupts normal cellular metabolism that results in the production of ROS. Plants have evolved diverse mechanisms to keep ROS homeostasis in cells, including antioxidative enzymes (e.g., SOD and CAT) and chemical antioxidants (e.g., glutathione and ascorbate) (Figure 3A). Among them, SOD acts as the first line of defense by converting O_2_^●−^ into H_2_O_2_, and CAT converts H_2_O_2_ into H_2_O and O_2_. They are both involved in plants’ drought tolerance. For example, the transgenic alfalfa expressing *MnSOD* gene from *Nicotiana plumbaginifolia* improved its survival and vigor after exposure to water deficit [127]. Overexpression of a cytosolic copper-zinc SOD from the mangrove plant *Avicennia* in rice can enhance its drought tolerance [128]. In addition, the increase of CAT activity is positively related to the drought degree [129], although the functional analysis of *CAT* gene on drought tolerance in transgenic plants is still scarce. The diverse abundances of SODs indicated that their functional state is dependent on subcellular location (e.g., plastid, peroxisome, or cytosol), drought conditions, and the plant’s drought adaptation ability. For example, the cytosolic Cu-Zn SODs were increased in two *O. sativa* cultivars (i.e., drought avoidance CT9993 and drought tolerance IR62266), while the chloroplast Cu-Zn SODs were increased in CT9993, but decreased in IR62266 [30]. Additionally, in a drought-sensitive cultivar of *Malus domestica*, the abundance of Cu-Zn SOD was decreased, but the FeSOD was increased [64].

POD catalyzes the reduction of H_2_O_2_ using various electron donors such as phenolic compounds, lignin precursors, auxin, and secondary metabolites. The drought-induced activities of POD were observed in leaves from *Arabidopsis* [130], *Ramonda serbica* [131], and *M. sativa* [132]. In proteomic studies, the abundances of PODs were increased in leaves of *Quercus* *ilex* [62] and a drought-tolerant *O. sativa* cultivar [32]. Moreover, the expression of POD genes was also increased in *Tamarix hispida* under drought stress [133]. These results indicate that PODs are critical for ROS scavenging in drought-stressed plants.

### 5.2. Ascorbate-Glutathione (AsA-GSH) Pathway

The AsA-GSH pathway is another key antioxidant pathway in response to drought [134,135]. During this process, the ascorbate peroxidase (APX) reduces H_2_O_2_ to H_2_O using ascorbate (AsA) as an electron donor, then the oxidized AsA is restored by monodehydroascorbate reductase (MDHAR), dehydroascorbate reductase (DHAR), and glutathione reductase (GR) [136] (Figure 3A). Proteomic studies revealed that most proteins in this pathway were increased under drought stress conditions. For example, the APXs localized in thylakoids and mitochondria were drought-increased in many herbaceous plants (e.g., *Cynodon* *dactylon* [19], *G. max* [17], *T. aestivum* [35,37,38], *O. sativa* [43,44,46], *H. vulgare* [37], and *B. napus* [47]). The function of APX under drought stress has been well addressed [129]. APX1-deficient mutant (*apx1*) of *A. thaliana* was significantly sensitive to drought stress [137]. Transgenic tobacco plants overexpressing cytosolic APX alleviated the damage from water stress. In addition, rice *Osapx2* mutants had lower APX activity and were sensitive to drought, whereas overexpression of *Osapx2* in rice enhanced its stress tolerance [138].

Another AsA-GSH pathway-related enzyme, GR, catalyzes the reduction of glutathione disulfide (GSSG) to the sulfhydryl form GSH. Consistently with the drought-increased abundance of GR, the drought-induced *GR* genes were also found in cowpea and *P. vulgaris* under drought stress [139,140]. Overexpression of *B. rapa* *BrGR* in *E. coli* showed an increase of GR activity and tolerance to H_2_O_2_ [141]. Thus, the positive function of GR in plant drought tolerance can be presumed [142]. Besides, two enzymes that can maintain ascorbate in its reduced state, dehydroascorbate reductase (DHAR) and monodehydroascorbate reductase (MDHAR), were found to be increased in some plants (i.e., *C. dactylon* and *O. sativa*) under drought stress. The expression of DHAR is generally associated with plant drought tolerance [143,144]. Overexpression of DHAR has been shown to increase drought tolerance or biomass in transgenic potato [145], rice [146], and tobacco [147]. These imply that DHAR can be a very promising target to improve plant drought stress tolerance.

In addition, proteomic studies revealed that some proteins were involved in glutathione-mediated ROS scavenging: glyoxalase (GLO), phospholipid hydroperoxide glutathione peroxidase (PHGPX), glutamate-cysteine ligase (GCL), glutaredoxin (Grx), and monothiol Grx. GLO catalyzes the detoxification of methylglyoxyl, which is involved in the indirect scavenging of ROS. Among them, the abundances of GLOs were not always increased in plants under drought stress. Their response to drought was dependent on the plant genotype and duration of drought. Besides, GCL is the first enzyme in the GSH biosynthetic pathway which was increased in drought-stressed *B. napus* [47]. Similar profiles were also observed in monothiol Grx in *B. napus* [47], *Z. mays* [41], as well as PHGPX in *Citrullus* *lanatus* [48]. All these suggest that the GSH-mediated antioxidative defense pathway is increased in leaves as a drought adaptation mechanism.

### 5.3. Peroxiredoxin/Thioredoxin (Prx/Trx) and Glutathione Peroxidase/Glutathione S-Transferase (GPX/GST) Pathway

The Prx/Trx pathway plays a central role in detoxification of H_2_O_2_. In this process, Prx catalyzes the reduction of H_2_O_2_, and then uses Trx to restore its catalytic activity (Figure 3A). The abundances of Prx and Trx were increased in response to drought, indicating the enhanced detoxification in drought-stressed leaves. Besides, a Trx-linked enzyme, methionine sulfoxide reductase (MSR), was also increased in some plants [57]. MSR is involved in the enzymatic conversion of methionine sulfoxide to methionine, which is involved in the protection of cells and tissues from H_2_O_2_-induced stress [148]. It has been reported that various oxidative stresses, including drought stress, can enhance the expression of numerous *Msr* genes [149]. This implies that MSR plays an important role in response to drought stress.

Besides, the GPX/GST pathway is enhanced in some plants under water deficit conditions. GPX catalyzes the reduction of H_2_O_2_ using Trx [150], and GST catalyzes conjugation reactions between GSH and a number of xenobiotics, playing a crucial role in the degradation of toxic substances. Previous studies have reported that overexpression of the *GST* gene enhanced drought tolerance in tobacco [151,152] and *Arabidopsis* [153]. Similarly, overexpression of *P. glaucum* *GPX* in rice resulted in an increased tolerance to drought stress [154]. In proteomic studies, the GPXs were drought-increased in *Boea* *hygrometrica* [21], *E. elongatum* [20], and *B. napus* [47], while drought-increased GSTs were widely found in the plant species discussed in this review. The increases of GPX and GST reinforced the functional evidence for their potential detoxification role in drought tolerance.

## 6. Pathogenesis Related Proteins

In proteomics results, several pathogenesis-related proteins were increased in response to drought stress, such as chitinase, disease resistance protein (DRP), polyphenol oxidase (PPO), oryzacystain, pathogen defense-related protein 10 (PR10), and disease resistance gene analog PIC15. Chitinase functions in the determent of herbivory and pathogen attack by acting on insect exoskeletons and fungal cell walls [155]. Five basic chitinase species and an acidic chitinase were increased in two tree species, i.e., *Eucalyptus* sp. [55] and *Musa* *paradisiaca* [42], respectively. The induced transcript of basic chitinase was also observed in *Eucalyptus* sp. [55]. Besides, PPO catalyzes the oxygen-dependent oxidation of phenols to quinones during plant defense against pests and pathogens. It was reported that the abundances and activities of PPOs were also increased in leaves of *B. hygrometrica* under desiccation [21], indicating its substantial role in the adaptation to severe water deficit. Moreover, an oryzacystain was increased in drought-stressed *O. sativa* leaves. Oryzacystain is a cysteine proteinase inhibitor in the phytocystatin family of proteinase inhibitors, which plays an important role in plant defense against pathogen attacks and oxidative stress [156]. The tobacco overexpressing the oryzacystain gene displayed an increase of drought tolerance by improving total SOD and guaiacol POD activities [157]. In addition, the drought-induced PR10 and disease resistance gene analog PIC15 were also found in leaves (Supplementary Table S1). It was found that overexpression of *PR10* can improve drought tolerance in tobacco [158]. The response of these pathogenesis-related proteins to drought suggests that there is a cross-talk between biotic and abiotic stresses.

## 7. Osmotic Regulation

Osmotic regulation is crucial for plant drought resistance. Several important osmotic homeostasis-related proteins, such as late embryogenesis abundant (LEA) protein, dehydrin (DHN), and betaine aldehyde dehydrogenase (BADH), were accumulated in leaves under drought stress. Among them, LEA proteins are highly hydrophilic proteins which function in plant abiotic stress as cellular protectants to stabilize cellular components in response to water loss [159]. Similarly, in physiological studies [160], DHNs (also known as group 2 LEA proteins) were widely drought-accumulated in several plant species, including *Z. mays* [41], *C. dactylon* [19], *T. aestivum* [35], and *B. napus* [47]. Higher hydrophilicity and thermostability of DHNs suggest that they can stabilize the protein structure through detergent- and chaperone-like properties [161]. Besides, the DHN in *Z. mays* exhibited a significantly increased phosphorylation level under drought stress [43]. The phosphorylation of LEA2 may facilitate its binding to calcium. LEA2 acts as calcium buffer, and has calcium-dependent chaperone-like activity, which is similar to that of calreticulin and calnexin [162]. Besides, group 3 LEA proteins were also increased in *Z. mays* [41] and *B. napus* [47] under certain drought conditions. It has been reported that transformation of the *LEA* gene into a number of plant species can confer tolerance to drought stress. For example, the transgenic calli overexpressing sweet potato *LEA14* (*IbLEA14*) enhanced the plant tolerance to drought stress, whereas RNA interference (RNAi) calli exhibited increased stress sensitivity [163]. All these indicate that *LEA* can be taken as a candidate gene for improving plant drought tolerance. Another osmotic regulation-related enzyme, BADH, showed drought accumulation in leaves of *H. vulgare* [27] and *Hordeum* *spontaneum* [25]. BADH converts betaine aldehyde to glycine betaine, which mediates osmotic homeostasis, and has positive effects on plant adaptation to drought stress [164]. RNAi-directed downregulation of *OsBADH1* resulted in a decrease of stress tolerance and an increase of oxidative stress [165], implying its important role in plant drought tolerance.

## 8. Modulation of Cell Structure and Cell Cycle

### 8.1. Cytoskeleton and Cell Cycle

In leaves, water deficit rapidly decreases cell division rate [166,167]. Proteomic studies revealed that the abundances and phosphorylation levels of several proteins involved in cell division (i.e., cell division cycle protein, division protein ftsZ1, and cyclin A2) were decreased under water deficit [22,28,44,47] (Figure 3C). Because cytokinesis requires new cytoskeleton and cell wall components, it is predictable that the cell wall and cytoskeleton-related proteins would change in response to drought. The cytoskeleton (i.e., microtubules and actin filaments) is a highly dynamic component, which is crucial for cell division, movement, morphogenesis, and signal transduction [168]. In proteomic studies, several cytoskeleton proteins, such as actin, kinesin motor protein, tubulin, profilin, actin depolymerizing factor, and fibrillin were decreased in response to drought stress (Figure 3B). The *actin* genes were also reduced in drought-treated *H. vulgare* leaves [168]. The decrease of the cytoskeleton and cell cycle-related proteins implies that cell growth is suppressed during drought stress.

In response to drought, the translationally-controlled tumor protein homolog (TCTP) was significantly drought-increased in *H. vulgare* [26], *T. aestivum* [37], and *B. napus* [47], which would facilitate plant adaptation to drought stress. TCTP is a Ca^2+^-binding protein with important functions in some cellular processes (e.g., protection against stress and apoptosis, cell growth, cell cycle progression, and microtubule organization) [169]. The protein abundance and gene expression of TCTP were increased in *P. indica*-colonized plants under drought stress. Additionally, PEG treatment for 48 h also increased the abundance of TCTP in leaves of *T. aestivum* [37]. Overexpression of *Arabidopsis* TCTP enhanced drought tolerance by rapid ABA-mediated stomata closure [170], while a TCTP knockdown mutant of *Arabidopsis* showed severe growth defects [171]. These results imply the importance of TCTP for plant drought tolerance.

### 8.2. Cell Wall Modulation

Water loss in plant tissues controls turgor pressure and directly affects the extensibility of the cell wall. Some drought-responsive enzymes involved in cell wall polysaccharide synthesis/hydrolysis, lignin biosynthesis, and cell wall loosening in leaves have been identified in proteomic studies (Figure 3D). Two enzymes involved in cell wall polysaccharide synthesis, reversibly glycosylated polypeptide and pectinacetylesterase, were drought-increased in *M. sativa* [50] and *B. napus* [47], respectively. Two enzyme inhibitors involved in polysaccharide hydrolysis inhibition, the xylanase inhibitor and polygalacturonase inhibitor, were decreased in drought-sensitive *Z. mays* cultivars, but increased in leaves of a tolerant cultivar [41]. Their changes in response to drought stress were in contrast to that of glucan exohydrolase, which is an enzyme involved in polysaccharide hydrolysis. These results indicate that the cell wall synthesis is enhanced in a drought-tolerant maize cultivar under drought stress, which may be associated with drought adaptation. Besides, three lignin biosynthesis related proteins, phenylalanine ammonia-lyase (PAL), caffeic acid 3-*O*-methyltransferase, and caffeoyl-CoA *O*-methyl-transferase, were generally increased under drought stress (Figure 3D). PAL catalyzes the transformation of phenylalanine to cinnamylate in the first step of lignin biosynthesis [172]. The activity of PAL was also obviously increased in the leaves of *Trifolium repens* under the early stages of drought stress (0–14 days), but decreased gradually as the period of stress was extended [173]. In addition, two drought-increased cell wall structural proteins (i.e., glycine-rich protein and fasciclin-like arabinogalactan protein) were found in the leaves of *B. napus* [47], which would enhance cell wall synthesis in response to drought stress. Interestingly, a significantly increased abundance and phosphorylation level of sucrose synthase was found in *Z. mays* [27,32,41,44], which would facilitate the synthesis of cell wall components by providing UDP-glucose directly to the cellulose synthases and/or callose synthases [174]. The drought-increased cell wall synthesis would enhance the mechanical strength for minimizing water loss and cell dehydration, which is crucial for plants to resist and recover from drought.

Besides, cell wall loosening is also important for growth adaptation in expanding leaves [166]. Two cell wall loosening/expansion-related enzymes, polygalacturonase/pectin depolymerase (PG) in *O. sativa* [29] and xyloglucan endotransglycosylase (XTH) in *Z. mays* [41], were increased under drought stress. PG can degrade pectin, while XTH can cleave and reform the bonds between xyloglucan chains to regulate cell wall rigidity. Previous studies have found drought-induced *XTH* genes upregulated in *Arabidopsis* and rice [175,176], which implies that XTH might serve as a stress marker gene in leaves. Taken together, the cell wall modulation, which results in either growth arrest in drought-sensitive cultivars or a continuation of growth with a reduced rate [177], would contribute to drought adaption by cell size adjustment for cell turgor maintenance [17].

## 9. Membrane Trafficking

Plant membrane transport system plays a significant role in response to water scarcity. In proteomic studies, several membrane trafficking proteins localized in mitochondrion, plasma, and vacuole were changed in response to drought. Two mitochondrial carrier proteins (i.e., dicarboxylate/tricarboxylate carrier (DTC) and 2-oxoglutarate/malate carrier protein (OMC)) were decreased in *T. aestivum* [35] and *G. max* [17], respectively. DTC and OMC can catalyze the transport of various metabolites (e.g., dicarboxylates, tricarboxylates, amino acids, and keto acids) across the inner mitochondrial membrane, and play an important role in several metabolic processes, such as the gluconeogenesis, nitrogen metabolism, as well as biotic stress [178]. However, three voltage-dependent anion channel proteins (VDAC) in *T. aestivum* [35] and two mitochondrial outer membrane porin 1-like proteins in *B. napus* [47] were significantly increased under drought stress. The VDAC localized in the outer membrane of mitochondria can regulate Ca^2+^ fluxes, ATP/ADP exchange, and metabolites. The abundance changes of these mitochondrion transport-related proteins under drought stress indicated that the ion/metabolite exchange between mitochondria and cytosol was modulated in leaves to cope with the stress. Among them, the function of VDAC has been proved to be involved in plant drought tolerance [179,180]. The transcriptional level of *VDAC* was induced in *Brassica rapa* by drought stress [179], and the overexpression of the *AtVDAC2* gene can confer drought resistance in *Arabidopsis* [180].

Remorin is a plant-specific plasma membrane protein that plays important role in plant-microbe interaction and signal transduction [181]. Some drought-increased remorins were found in *Z. mays* [41], *O. sativa* [44], and tolerant cultivars of *T. aestivum* [35]. Overexpressing heterologous remorin in *Arabidopsis* enhanced the tolerance to dehydration and salinity at the germination and seedling stages [182]. Besides, aquaporin (AQP), which transports water and other small molecules, is crucial for plants to combat drought stress. PEG treatment upregulated the expression of a PIP2 subgroup gene, *AQP* (*TaAQP7*), and the overexpression of *TaAQP7* increased drought tolerance in tobacco [183]. Similarly, a proteomic study also found an increased abundance of AQP in *Z. mays* under 12.5% soil water stress for 6 days [41]. Additionally, drought-induced abundance of lipid transfer protein (LTP) was observed in leaves of *C. albidus* [53]. LTP has been proved to be involved in abiotic stress [184]. In *Arabidopsis*, the loss-of-function mutant of LTP3 was sensitive to drought stress, whereas overexpressing plants appeared drought tolerant [185]. Moreover, the *OsDIL*-overexpressing transgenic *O. sativa* plants were more tolerant to drought stress during vegetative and reproductive development [186]. All these demonstrated that LTP was involved in plant tolerance to drought stress, and is probably a candidate gene for genetic improvement of crop yield in adaption to drought stress.

In addition, vacuolar H^+^-pyrophosphatase (V-PPase), vacuolar-ATPase (V-ATPase), and ABC transporter ATPase showed dynamic changes in response to drought. V-ATPase and V-PPase are two tonoplast proton pumps for translocating H^+^ into the vacuoles to generate a gradient of H^+^, which provide a driving force for the accumulation of ions and other solutes in the vacuole. Their functions in plant abiotic stress tolerance have been widely discussed [187,188]. For example, an *M. domestica* vacuolar H^+^-ATPase (VHA) encoding gene (*MdVHA-A*) was induced in shoots under PEG treatment, and overexpression of *MdVHA-A* in transgenic tobacco seedlings improved drought tolerance [189]. In *Z. mays*, the abundances of V-ATPase and V-PPase were increased in a drought tolerant cultivar, but decreased in an intolerant one [41]. The differential expression patterns of ATPase and V-PPase further indicate their important functions in plants’ response to osmotic stress. Taken together, all the changes of membrane trafficking-related proteins in mitochondrion, plasma, and vacuole highlight that ion transport and membrane trafficking are crucial in leaves in order to cope with water deficit.

## 10. Photosynthesis and Photorespiration

**Figure 4:**
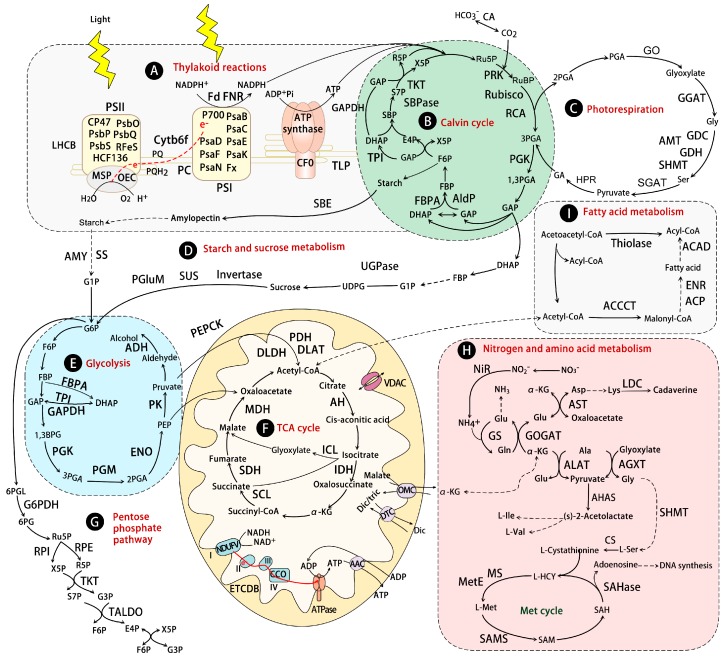
Schematic model of drought responsive proteins involved in photosynthesis, carbon metabolism, fatty acid metabolism, and nitrogen and amino acid metabolism. The solid line indicates single-step reactions, and the dashed line indicates multi-step reactions. (**A**) Thylakoid reaction; (**B**) Calvin cycle; (**C**) Photorespiration; (**D**) Starch and sucrose metabolism; (**E**) Glycolysis; (**F**) Tricarboxylic acid (TCA) cycle; (**G**) Pentose phosphate pathway; (**H**) Nitrogen and amino acid metabolism; (**I**) Fatty acid metabolism. 1,3BPG: 1,3-bisphosphoglycerate; OMC: 2-oxoglutarate/malate carrier protein; MetE: 5-methyltetrahydropteroyltriglutamate-homocysteine methyltransferase; 6PG: 6-phosphogluconate; G6PDH: 6-phosphogluconate dehydrogenase; 6PGL: 6-phosphoglucono-δ-lactone; AHAS: acetohydroxyacid synthase; AH: aconitate hydratase; ACAD: acyl-CoA dehydrogenase; SAHase: adenosylhomocysteinase; ALAT: alanine aminotransferase; AGXT: alanine-glyoxylate aminotransferase; ADH: alcohol dehydrogenase; AldP: aldolase; AMT: aminomethyl transferase; AMY: amylase; AST: aspartate aminotransferase; ATPase: ATP synthase; AAC: ATP/ADP translocase; CA: carbonic anhydrase; FNR: chloroplast ferredoxin-NADP^+^ oxidoreductase; MSP: chloroplast manganese stabilizing protein; CS: cysteine synthase; Cyt b6f: cytochrome b6f complex; CCO: cytochrome c oxidase; Dic: dicarboxylate; DTC: dicarboxylate/tricarboxylate carrier; DLAT: dihydrolipoamide acetyltransferase; DHAP: dihydroxyacetone phosphate; ETCDB: electron carrier/iron ion binding protein; ENO: enolase; E4P: erythrose-4-phosphate; Fd: ferredoxin; F6P: fructose 6-phosphate; FBP: fructose-1,6-bisphosphate; FBPA: fructose-bisphosphate aldolase; G6P: glucose 6-phosphate; G1P: glucose-1-phosphate; GOGAT: glutamate synthase; GGAT: glutamate-glyoxylate aminotransferase; GS: glutamine synthetase; GAP: glyceraldehyde 3-phosphate; GAPDH: glyceraldehyde-3-phosphate dehydrogenase; GA: glyceric acid; GCS: glycine cleavage system; GDC: glycine decarboxylase; GO: glycolate oxidase; HCY: homocysteine; HPR: hydroxypyruvate reductase; IDH: isocitrate dehydrogenase; ICL: isocitrate lyase; LHCB: light-harvesting chlorophyll a/b-binding protein; LDH: lipoamide dehydrogenase; LDC: lysine decarboxylase; MDH: malate dehydrogenase; MS: methionine synthase; NDUFV: NADH dehydrogenase (ubiquinone) flavoprotein; NIR: nitrite reductase; OEC: oxygen-evolving complex protein; PEPCK: phosphoenol pyruvate carboxykinase; PEP: phosphoenolpyruvate; PGluM: phosphoglucomutase; PGA: phosphoglycerate; PGK: phosphoglycerate kinase; PGM: phosphoglycerate mutase; PRK: phosphoribulokinase; PSI: photosystem I reaction center protein; PSII: photosystem II reaction center protein; PC: plastocyanin; PDH: pyruvate dehydrogenase; DLDH: pyruvate dehydrogenase complex; PK: pyruvate kinase; R5P: ribose-5-phosphate; RPI: ribose-5-phosphate isomerase; Ru5P: ribulose 5-phosphate; Rubisco: ribulose-1,5-bisphosphate carboxylase/oxygenase; RCA: ribulose-1,5-bisphosphate carboxylase/oxygenase activase; RuBP: ribulose-1,5-disphosphate; RPE: ribulose-phosphate 3-epimerase; SAH: *S*-adenosyl-l-homocysteine; SAM: *S*-adenosylmethionine; SAMS: *S*-adenosylmethionine synthase; S7P: sedoheptulose 7-phosphate; SBPase: sedoheptulose-1,7-bisphosphatase; SBP: sedoheptulose-1,7-bisphosphate; SGAT: serine glyoxylate aminotransferase; SHMT: serine transhydroxymethyltransferase; SBE: starch branching enzyme; SS: starch synthase; SDH: succinate dehydrogenase; SCL: succinyl-CoA ligase; SUS: sucrose synthase; TLP: thylakoid lumen protein; TALDO: transaldolase; TKT: transketolase; Tric: tricarboxylate; TPI: triosephosphate isomerase; UDPG: uridine diphosphate glucose; UGPase: UTP-glucose pyrophosphorylase; VDAC: voltage dependent anion channel; X5P: xylulose 5-phosphate; α-KG: α-ketoglutarate.

It is well known that photosynthetic inhibition is one of the primary detrimental effects of water stress due to stomatal closure [190,191]. Thus, it is predictable that the universal decrease trends of photosynthesis-related proteins would be found in drought-stressed leaves (Figure 4A,B). Plants have developed many strategies to cope with drought stress, one important aspect is the recovery of photosynthesis. The drought-increased proteins involved in photoreaction and Calvin cycle were observed in leaves. For example, light-harvesting chlorophyll a/b-binding proteins (LHCB) were increased in tolerant genotypes of *Z. mays* [41], and *M. domestica* [64], but decreased or stable in sensitive genotypes. The LHCBs have been predicted to be involved in ABA signaling partially by modulating ROS homeostasis [192], and may be taken as interesting targets for crop breeding [4]. Besides, the increased abundance of sedoheptulose-1,7-bisphosphatase (SBPase) and carbonic anhydrase (CA) were found in drought-stressed *P. pratensis* [23] and *M. domestica* [64]. SBPase has a key role in regulating the photosynthetic Calvin cycle. CA catalyzes the reversible hydration of CO_2_, and has a relevant role in CO_2_ exchange by influencing the internal conductance [193].

In addition, the abundances of proteins involved in photorespiration were also decreased in some plant species, reflecting the drought-inhibited photorespiration process (Figure 4C). However, significant increases of four key enzymes (e.g., glycolate oxidase, glycine dehydrogenase, serine glyoxylate aminotransferase, and serine transhydroxymethyl transferase) were found in a high water-use efficiency *M. domestica* variety under moderate drought conditions [64]. Additionally, the protein abundances of aminomethyl transferase (AMT) and glycine dehydrogenase were decreased in a drought-sensitive cultivar of *C. dactylon*, but increased or stable in a tolerant cultivar [19]. Similarly, the downregulation of the gene encoding AMT was reported in dehydration-sensitive *H. vulgare*, but not in a dehydration-tolerant cultivar [194]. It is well known that photorespiration can protect photosynthesis from photoinhibition and prevent ROS accumulation in green tissues [195]. The proteomic findings provided further evidences that the regulation of photorespiration was important for plant drought tolerance [64].

## 11. Carbohydrate and Energy Metabolism

Proteomic studies have revealed that nearly 20% of total drought-responsive proteins were involved in carbohydrate and energy metabolism (e.g., glycolysis, tricarboxylic acid (TCA) cycle, electron transport chain, and ATP synthesis) in leaves to cope with drought stress (Figure 4E–G). For example, phosphoglucomutases (PGluMs) involved in glycolysis/gluconeogenesis were decreased in leaves of *S. stapfianus* [24] and *O. sativa* (stop irrigation for 35 days) [32], but increased in leaves of *C. lanatus* [48], *O. sativa* (under 30% relative humidity treatment for 144, 168 and 192 h) [29], and *M. paradisiaca* [46] in response to drought. Fructose-bisphosphate aldolase (FBPA), another glycolysis-related protein, was decreased in many aforementioned plant species, but also found to be drought-increased in other plant species such as *O. sativa* [29,32], *S. stapfianus* [24], and *M. paradisiaca* [46]. Besides, FBPA abundance was decreased in drought-sensitive cultivars of *M.* *domestica* [64] and *P. pratensis* [23], but was increased in tolerant cultivars. Similarly, aconitate hydratases involved in the TCA cycle were increased in leaves of tolerant cultivars of *Z. mays* and *P. pratensis* [23], whereas they were decreased in sensitive cultivars of *Z. mays* and *P. pratensis* [23]. The inhibition would be a mechanism for accumulating sugars as an osmolyte or energy source for recovery [62], while the increase of glycolysis and TCA may act as a strategy for providing energy during the activation of stress defenses, especially when the photosynthesis was inhibited. These apparently contradictory results could also be related to the different degrees and duration of water shortage, as well as to the tolerance ability of the plant species [62]. Among these proteins, cytosolic FBPA and glyceraldehyde-3-phosphate dehydrogenase (GAPDH) have a significantly positive correlation with drought tolerance [196], and overexpressing GAPDH in potato displayed an improvement of drought tolerance [197]. In addition, the reverse phosphorylation of some carbohydrate metabolism-related proteins (e.g., phosphoenolpyruvate carboxykinase [44], FBPA [44], and NAD-malate dehydrogenase (MDH) [34]) (Supplementary Table S2) further demonstrated that the carbon metabolism was modulated in plants in response to water stress. Although many drought-responsive carbohydrate metabolism-related proteins were revealed by proteomic studies, only a few of them have been functionally characterized to evaluate their role in drought stress tolerance.

Consistent with carbohydrate metabolism changes, mitochondrial electron transport chain and ATP synthesis-related proteins were changed in plants under drought stress. They were comprised of ATP synthase subunits (e.g., ATPase F0, F1, α, β, γ, δ, and ε subunits) and electron transport chain-related proteins (NADH dehydrogenase [47,86], quinone oxidoreductase [35,49,62], and cytochrome c oxidase [18,39,47]). Their changes imply that diverse changes of energy metabolism take place in leaves in response to water deficit. In some cases, plants may have the ability to enhance energy production to maintain major physiological activity and inhibit stress damage.

## 12. Nitrogen Assimilation and Amino Acid Metabolism

Nitrogen assimilation is affected by abiotic stress in plants. In this process, exogenous absorbed nitrate is transformed to ammonium by nitrate reductase (NR) and nitrite reductase (NIR), and then assimilated by glutamine synthetase (GS) and glutamate synthase (GOGAT) into amino acids. Proteomic studies revealed that several enzymes in nitrogen assimilation were drought-decreased in leaves (Figure 4H). Long-term (3 weeks) drought stress decreased the abundance of NIR in *H. vulgare* [25], but short-term (2–5 days) drought stress increased NIR in *O. sativa* [29]. Besides, drought significantly decreased the protein abundances of GS1, GS2, and GOGAT in many plant species (Supplementary Table S1), and the activities of NR and GS were reduced in *Leymus chinensis* [198], *T. aestivum* [199], and tomato [200] under water deficit. Similarly, the decrease at transcriptional level of *Brassica juncea GS1.1* and other key genes involved in nitrogen assimilation (e.g., *BjGDH1*, *BjGDH2*, and *BjASN2*) were also well addressed [201]. The reduction of NR, GS, and GOGAT indicates that the nitrogen assimilation is significantly inhibited by drought stress, which is supposed to be the main reason for the reduction of crop yield under drought stress.

Following the GS/GOGAT cycle, the amino group was transferred into other amino acids by aminotransferases (Figure 4H). Two aminotransferases (e.g., aspartate aminotransferase (AST) and alanine aminotransferase (ALAT)) were altered under drought stress. Among them, AST was drought-decreased in *P. pratensis* [23], and ALAT was decreased in *P. pratensis* [23] and *Z. mays* [41]. AST catalyzes the amino transfer from glutamate to oxaloacetate to form aspartate, which is part of ammonium assimilation. ALAT catalyzes the interconversion of l-glutamate and l-alanine. The abundance decline of these enzymes indicates that the amino acid metabolism and the synthesis of other metabolites derived from amino acids are inhibited under drought stress.

Interestingly, several key enzymes involved in *S*-adenosyl-l-methionine (SAM) cycle were generally increased in leaves in response to drought stress, including drought-increased 5-methyltetrahydropteroyltriglutamate-homocysteine methyltransferase (MetE), *S*-adenosyl-l-homocysteine hydrolase (SAHase), *S*-adenosylmethionine synthase (SAMS), and methionine synthase (MS) (Figure 4H). Similarly, MS in *Z. mays* [41], *Sorghum bicolor* [202], a drought-tolerant *C. dactylon* cultivar, as well as SAMS in *Populus* [59,86], *Q. ilex* [63], and *T. aestivum* [35] were increased under drought conditions. The metabolites in the SAM cycle were known to play major roles in methylation of DNA, proteins, and other metabolites, being involved in control of gene expression, cell wall metabolism, as well as the biosynthesis of polyamine and ethylene for stress tolerance [18,203,204]. In addition, the *MS* gene was also strongly induced in barley leaves under drought stress [205]. Transgenic *Arabidopsis* lines overexpressing *SAMS* from *Solanum brevidens* enhanced the ability of plant drought tolerance [206]. This implies that the increases of MS and SAMS could enhance the methionine and osmoregulant metabolism for plants to cope with drought stress.

## 13. Fatty Acid Metabolism and Other Metabolisms

Four proteins involved in fatty acid biosynthesis (i.e., acetyl-coenzyme A carboxylase carboxyl transferase, acyl carrier protein, enoyl-acyl carrier protein reductase, and lipoxygenase 6) and three involved in fatty acid degradation (i.e., thiolase I, thiolase II, and acyl-CoA dehydrogenase) were altered under dehydration conditions (Figure 4I). Physiological and molecular investigations have revealed that the fatty acid/lipid metabolism was changed in response to drought stress [207,208,209]. For example, a greater composition of unsaturated fatty acids in membrane lipids may contribute to superior leaf dehydration tolerance in *C. dactylon* [209]. In the leaves of *Parkinsonia aculeata*, the significantly increased contents of chloroplast lipids (i.e., monogalactosyldiacylglycerol and digalactosyldiacylglycerol) and extra-plastidial lipids (i.e., phosphatidylcholine and phosphatidylethanolamine) maintained *Parkinsonia* plants in a state of rapid recovery of lipid synthesis during rehydration [209]. The changes in fatty acid/lipid composition may help to maintain membrane integrity and preserve cell compartmentation under water stress [210].

In addition, two flavonoid biosynthesis related proteins (i.e., chalcone isomerase (CHI) and dihydroflavonol-4-reductase) involved in secondary metabolism were also changed in response to drought (Supplementary Table S1). It has been suggested that flavonoids have antioxidant capacity to protect plants against abiotic stress [211,212], and flavonoid accumulation was induced rapidly by abiotic stresses [213]. In proteomic studies, CHI, a key enzyme in flavonoid biosynthesis, was decreased in *O. sativa* under drought stress [29]. Similarly, the *CsCHI* gene in leaves of tea (*Camellia sinensis*) was also downregulated under drought treatment [214]. Besides, dihydroflavonol-4-reductase, another key enzyme in the flavonoid biosynthesis pathway, was decreased in a drought sensitive *Z. mays* cultivar [41], but increased in a tolerant one. The abundance changes of these enzymes currently attract great attention from researchers seeking to evaluate their potential protective roles in drought stress response.

## 14. Concluding Remarks

**Figure 5:**
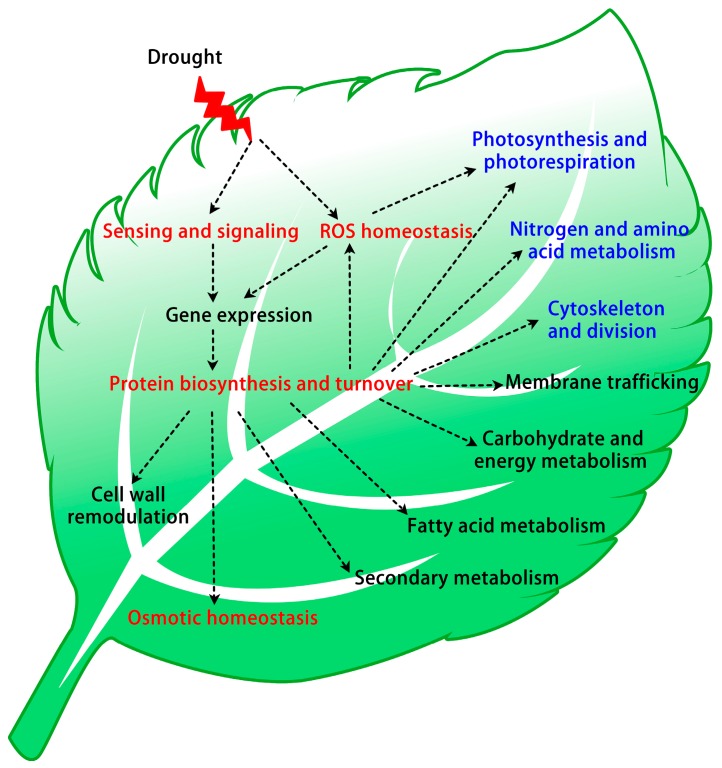
A summary of signaling and metabolic pathways in leaves in response to drought stress. Drought stress activates several signaling cascades, regulates gene expression, and promotes protein biosynthesis and turnover in drought-stressed leaves. Besides, drought stress inhibits photosynthesis, photorespiration, nitrogen and amino acid metabolism, as well as the cytoskeleton and cell division. Importantly, plants enhance ROS scavenging, osmotic regulation and cell wall remodulation. In addition, plants also modulate membrane trafficking, fatty acid metabolism, carbohydrate and energy metabolism, as well as secondary metabolism to cope with drought stress.

By integrative analysis of proteomics results, it has been found that, in total, 2216 protein species and more than 200 phosphoproteins were drought-responsive in plant leaves. The abundance changes of these proteins provide new clues towards further understanding the complex cellular and molecular processes in plant drought tolerance. The quantitative patterns of protein in various plants give rise to diverse drought-responsive mechanisms, including drought signal transduction, ROS scavenging, osmotic regulation, specific gene expression regulation, protein synthesis and turnover, cell structure modulation, as well as carbohydrate and energy metabolism (Figure 5). Exploring these protein functions will enable a holistic understanding of plant-environment interaction. Moreover, the potential proteins screened out by proteomics will provide candidate genes/proteins for genetic improvement in plant drought tolerance. However, some proteins showed dynamic changes depending on plant species and stress intensity, which makes it difficult to give a clear interpretation of the mechanism in plant drought response. Importantly, proteomic investigations also revealed that protein PTMs are crucial for signaling and metabolic mechanisms in plant drought response. The integration of those findings from physiological, gene expression, and other large-scale “omics” will help us to establish molecular networks of drought response and tolerance.

## Figures and Tables

**Table 1:** Summary of current publications on drought-responsive proteomics in leaves.

Plant Types	No.	Plant Species	Drought Treatment Condition	Protein Species	Unique Proteins	Reference
Herbs	1	*Agrostis stolonifera*	35% soil water	81	57	[18]
	2	*Cynodon dactylon*	withholding irrigation, 15 days	54	42	[19]
	3	*Elymus elongatum*	75%, 50%, 25% field capacity, rewater 3, 14 days	11	9	[20]
	4	*Boea hygrometrica*	50% relative humidity (0.5, 8, 48 h); rehydrated 8, 48 h	8	8	[21]
	5	*Poa pratensis*	TE foliar spray, 14 days; withholding irrigation, 0, 10 and 15 days	58	32	[22]
			withholding irrigation, 10 and 15 days	88	36	[23]
	6	*Sporobolus stapfianus*	30% relative water content	108	55	[24]
	7	*Hordeum spontaneum*	withhold water to −2.5 MPa soil water potential, 3 weeks	32	22	[25]
		*Hordeum vulgare*	*P. indica*-inoculation 28 days, 25% field capacity	45	28	[26]
			10% field capacity, 5 days	37	23	[27]
			stopping water, 7 days	24	23	[28]
	8	*Oryza sativa*	30% relative humidity, 48, 72, 96, 120, 144, 168 and 192 h	94	64	[29]
			withholding water, 38, 43 days; rewater 5, 10 days	17	13	[30]
			50% reposition of the water lost daily, 20 days	15	15	[31]
			stop irrigation, 35 days	53	31	[32]
			no irrigation, 20 days	20	18	[33]
			withholding water, 6 days	10	10	[34] *
	9	*Triticum aestivum*	without water 5, 14, 24 days; rewater 25 days	159	113	[35]
			1/3 of field capacity, 14 h	13	13	[36]
			PEG 6000, 48 h	23	19	[37]
			20%–25% soil relative water content, 3 days	29	16	[38]
			withholding water, 9 days	30	21	[39]
			20% PEG for 48 h	31	29	[40] *
	10	*Zea mays*	12.5% volumetric soil water content, 6 days	220	142	[41]
			withholding water, 9 days	29	27	[42]
			withholding water for 1, 4–5 days	138	111	[43] *
			PEG solution (−0.7 MPa) for 8 h	149	41	[44] *
	11	*Saccharum officinarum*	10% PEG 6000,14 h	4	4	[45]
	12	*Musa paradisiaca*	0.21 M sorbitol, 48 days	24	20	[46]
	13	*Brassica napus*	no watering, 3, 7, 10 and 14 days	417	253	[47]
	14	*Citrullus lanatus*	no irrigation, 3 days	29	13	[48]
	15	*Glycine max*	10% PEG 6000 or exposed to drought conditions, 4 days	51	39	[17]
	16	*Phaseolus vulgaris*	no irrigation, 17 days	81	40	[49]
	17	*Medicago sativa*	water withholding, 7 days	29	20	[50]
	18	*Gossypium herbaceum*	35% relative water content	18	17	[51]
Shrubs	19	*Carissa spinarum*	25% relative humidity, 48, 120 h, rewater 24 h	23	12	[52]
	20	*Cistus albidus*	no irrigation, 107 days; rewater, 26 days	56	35	[53]
	21	*Hippophae rhamnoides*	25% field capacity, 20 days	13	11	[54]
Trees	22	*Eucalyptus* sp.	dry season, 6 months	46	19	[55]
	23	*Populus* spp.	25% field capacity, 18 days then rewater 28 days in glasshouse; no irrigation, 86 days in open field	33	29	[56]
			withholding water 4 and 7 days	13	5	[57]
			withholding water 8, 12 days; rewater 7 days	52	25	[58]
			120 ppb ozone, 13 h; 35% soil water, 7, 14, 21 and 28 days	25	19	[59]
			10% soil water content, 45 days	40	33	[60]
	24	*Quercus robur*	15% soil water content 3, 8, 56 and 65 days	41	28	[61]
		*Quercus ilex*	no irrigation 14 days; no irrigation 7 days, rewater 7 days	14	11	[62]
			not watering, 28 days	18	11	[63]
	25	*Malus domestica*	45%–55% field capacity	81	33	[64]

The references labelled with * are phosphoproteomic studies. The information of these phosphoproteins is listed in Supplementary Table S2. TE: trinexapac-ethyl; PEG: polyethylene glycol.

## Supplementary Materials

Supplementary materials can be found at www.mdpi.com/1422-0067/17/10/1706/s1.
